# Childhood height, adult height, and the risk of prostate cancer

**DOI:** 10.1007/s10552-016-0731-8

**Published:** 2016-03-07

**Authors:** Lise Geisler Bjerregaard, Julie Aarestrup, Michael Gamborg, Theis Lange, Anne Tjønneland, Jennifer L. Baker

**Affiliations:** Institute of Preventive Medicine, Bispebjerg and Frederiksberg Hospital, The Capital Region, Nordre Fasanvej 57, Hovedvejen, 2000 Frederiksberg, Denmark; Section of Biostatistics, Department of Public Health, University of Copenhagen, Øster Farimagsgade 5B, 1014 Copenhagen K, Denmark; Danish Cancer Society Research Center, Strandboulevarden 49, 2100 Copenhagen K, Denmark; Novo Nordisk Foundation Center for Basic Metabolic Research, Faculty of Health and Medical Sciences, University of Copenhagen, Nørre Allé 20, 2200 Copenhagen K, Denmark

**Keywords:** Adult, Body height, Child, Cohort studies, Growth, Prostate neoplasms

## Abstract

**Purpose:**

We previously showed that childhood height is positively associated with prostate cancer risk. It is, however, unknown whether childhood height exerts its effects independently of or through adult height. We investigated whether and to what extent childhood height has a direct effect on the risk of prostate cancer apart from adult height.

**Methods:**

We included 5,871 men with height measured at ages 7 and 13 years in the Copenhagen School Health Records Register who also had adult (50–65 years) height measured in the Danish Diet, Cancer and Health study. Prostate cancer status was obtained through linkage to the Danish Cancer Registry. Direct and total effects of childhood height on prostate cancer risk were estimated from Cox regressions.

**Results:**

From 1996 to 2012, 429 prostate cancers occurred. Child and adult heights were positively and significantly associated with prostate cancer risk. When adjusted for adult height, height at age 7 years was no longer significantly associated with the risk of prostate cancer. Height at 13 years was significantly and positively associated with prostate cancer risk even when adult height was adjusted for; per height *z*-score the hazard ratio was 1.15 [95 % confidence interval (CI) 1.01–1.32].

**Conclusions:**

The effect of height at 13 years on the risk of prostate cancer was not entirely mediated through adult height, suggesting that child height and adult height may be associated with prostate cancer through different pathways.

## Introduction

Globally, prostate cancer is the second most frequently diagnosed cancer, and it accounts for 15 % of all cancers in men [1]. Despite the frequency, the precise etiological factors have not been identified. Well-described risk factors for prostate cancer are limited to age, family history, and race [1]. Recently, a focus has been placed on adult obesity as a potential risk factor for prostate cancer [2, 3]. Additionally, adult height has a consistent, moderate, and positive association with prostate cancer [3, 4]. Previously, we showed that taller boys had a significantly increased risk of prostate cancer, suggesting that the origins of the adult association may lie earlier in life [5]. It is, however, not known whether childhood height and adult height affect prostate cancer risk through different pathways, or whether the effect of childhood height is mediated through adult height. Therefore, the aim of this study was to investigate whether and to what extent childhood height has a direct effect on the risk of prostate cancer apart from the effect of adult height.

## Materials and methods

### Data sources

Information on height at school ages was obtained from the Copenhagen School Health Records Register (CSHRR) [6]. The CSHRR is a population-based cohort containing computerized information on 372,636 children who were born 1930–1989 and who attended a private or public school in the Copenhagen municipality. From 2 April 1968 onwards, a unique personal identification number was assigned to all Danish residents [7]. Children who attended school in 1968 or later had their identification number recorded on their health card, while it was retrieved for children who left school prior to this time.

Information on adult height was obtained by linkage of children in the CSHRR to the Danish Diet, Cancer and Health (DCH) cohort [8] via the identification number. Individuals included in the DCH cohort were enrolled from December 1993 to May 1997. Inclusion criteria were being 50–64 years of age, born in Denmark, residing in the Copenhagen or Aarhus areas, and no diagnosis of cancer registered in the Danish Cancer Registry prior to enrollment. Of 79,728 men who were invited to participate, 27,178 accepted the invitation, filled in a questionnaire, and were interviewed about their diet and lifestyle [8].

Via the identification number, information on prostate cancer status was obtained from the Danish Cancer Registry. It was established in 1942 [9], and the use of multiple notifications from different data sources secures a high degree of completeness. Further, through manual quality controls, validation of information in complex cases, and a high proportion of morphologically verified tumors, the validity of information in the register is enhanced [9]. Prostate cancer was defined by the International Classification of Diseases 10 code C61. Vital status was obtained by linkage to the national Vital Statistics Register [7].

#### Anthropometric information

Childhood height was measured by school doctors or nurses, and these health examinations were mandatory and performed annually through 1983 and thereafter only at school entrance and exit unless the child had specific health requirements [6]. To account for increases in height during childhood and across birth years, height measurements were transformed to *z*-scores based upon internal sex-, age-, and birth cohort-specific references (five-year intervals) using the Lamda, Median, Sigma (LMS) method [10].

Height at age 50–65 years (referred to as adult height) was measured by a trained laboratory technician [8]. To facilitate comparisons of the strengths of the associations across ages despite different standard deviations (SD) for heights, adult height was transformed to *z*-scores based upon internal birth cohort-specific references (1930–1934, 1935–1939, 1940–1944, 1945–1947). Adult height was as childhood height normally distributed, and the *z*-scores were calculated as standard *z*-score = [(observed value − population mean)/SD] using all information available among DCH males identified in the CSHRR.

#### Study population

The starting population for this study was men included in the CSHRR with an identification number, who were born 1930–1947, and who did not die, emigrate, or become lost to follow-up before 1 May 1993 or age 50 years (entry date and age criteria in the DCH cohort). In the eligible population of 6797 men included in both the CSHRR and the DCH, 62 were excluded due to a diagnosis of any cancer prior to enrollment in the DCH study, seven men with missing information on adult height, and 857 without information on height either at 7 or 13 years of age were excluded. Follow-up started on December 1993 through May 1997 at ages 50–65 years, upon enrollment into the DCH study, and ended at the date of diagnosis of prostate cancer, death, emigration, loss to follow-up, or 31 December 2012, whichever came first.

#### Ethics

The project was approved by the Danish Data Protection Agency. According to Danish law, ethical approval is not required for purely register-based studies.

### Statistical analyses

Anthropometric characteristics (mean, SD, and median) were calculated for the included men. Correlation analyses between height at ages 7 and 13 years and adult height were performed, and the number of cases and incidence rate of prostate cancer were calculated in categories of height at each age.

Cox proportional hazards regression was used to examine associations between height *z*-scores and the risk of prostate cancer. Age was used as the underlying timescale, and the baseline hazard was stratified by birth cohort (1930–1934, 1935–1939, 1940–1944, 1945–1947).

We examined associations between height *z*-scores at the ages of 7 and 13 years and in adulthood with prostate cancer, individually. Next, we examined the associations between height *z*-scores at ages 7 and 13 years, respectively, and prostate cancer with adjustment for adult height. Finally, associations between adult height *z*-score and the risk of prostate cancer were examined with adjustment for childhood heights.

The proportion of the risk of prostate cancer associated with childhood height mediated through adult height was estimated by the difference in coefficient method on the log hazard ratio (HR) scale, i.e., mediated proportion = (log(HR_child_) − log(HR_child+adult_))/log(HR_child_) * 100 [11, 12]. Confidence intervals (CIs) for the mediated proportion were computed by nonparametric bootstrapping (i.e., random sampling with replacement from the original sample) using 5,000 replications each time computing the implied value for the mediated proportion.

The effect of change in height during childhood was examined in a model including height *z*-scores at both 7 and 13 years, with and without adjustment for adult height *z*-score. Potential interactions between height *z*-scores at ages 7 and 13 years, respectively, and adult height *z*-score as a categorical variable (<−0.68, −0.68 to 0.68 [reference], >0.68) on the risk of prostate cancer were investigated based on the likelihood ratio test. None of these tests were statistically significant (range for *p* values 0.67–0.99).

The linearity of the associations in all models was assessed by testing against a restricted cubic spline with four knots [13]. Potential interactions between height *z*-scores and year of birth (1930–1934, 1935–1939, 1940–1947) were tested using a likelihood ratio test in nested models with and without cross-product terms. The proportional hazards assumption underlying the Cox models was investigated by including a term in the model that allowed the effect of the height *z*-score to change with age at risk and testing whether the change in the HRs was significant. Significant deviations from linearity, modification by birth cohort, or violations of the proportional hazards assumption were not observed (not shown).

## Results

In total, 5,871 men were included in the study. During 88,559 person-years of follow-up (mean = 15.1 years, range 0.2–19.1 years), 429 (7.3 %) prostate cancer diagnoses were recorded from 1996 through 2012 at the ages of 54.6–81.3 years. Of these, 112 died during follow-up. Mean height in childhood and adulthood increased by birth cohort (Table 1).

**Table 1:** Height by age of measurement and by birth cohort among boys from the Copenhagen School Health Records Register who participated in the Danish Diet, Cancer and Health study as adults

Age (years)	Birth cohort	*N*	Height (cm)		
			Mean	SD	Median
7	1930–1934	1,035	120.6	5.0	120.5
	1935–1939	1,779	121.0	5.1	120.9
	1940–1944	2,584	121.9	5.0	121.9
	1945–1947	473	123.0	5.0	123.2
13	1930–1934	1,035	150.4	7.1	150.0
	1935–1939	1,779	152.1	7.2	151.9
	1940–1944	2,584	153.1	7.4	152.9
	1945–1947	473	153.9	7.5	153.6
50–65 (adult)	1930–1934	1,035	175.3	6.3	175
	1935–1939	1,779	176.4	6.6	176
	1940–1944	2,584	177.0	6.5	177
	1945–1947	473	178.1	6.2	178

*cm* centimeters, *SD* standard deviation

As expected, height at ages 7 and 13 years and adult height were all strongly and positively correlated. The correlation between height at 7 years and adult height (*r* = 0.77) was slightly stronger than between height at 13 years and adult height (*r* = 0.70). The strongest correlation was between height at 7 and 13 years (*r* = 0.87).

The incidence rate of prostate cancer increased across categories of height at age 7 and 13 years and in adulthood (Table 2). We detected positive and significant associations of similar size between height at ages 7 and 13 years, respectively, and the risk of prostate cancer (Table 3). Height in adulthood was also associated with a significantly increased risk of prostate cancer (Table 3). Although the height *z*-scores are birth cohort-specific, moving from a *z*-score of 0 to a *z*-score of 1 corresponds to ~5.0 cm at 7 years, ~7.3 cm at 13 years, and ~6.5 cm in adulthood (Table 1).

**Table 2:** Number of cases and incidence rate of prostate cancer by categories of height *z*-scores at ages 7 and 13 years and in adulthood

Age (years)	Category of height *z*-score	Cm equivalent^a^	Frequency (%)	Cases (*n*)	Person-years of follow-up	Incidence rate per 1,000
7	−4.5 to −0.68	102.6–119.2	25.12	87	22,538	3.86
	−0.68 to 0.68	116.9–126.2	50.79	230	44,781	5.14
	0.68 to 4.5	124.0–138.1	24.08	112	21,240	5.27
13	−4.5 to −0.68	127.8–148.5	24.34	80	21,795	3.67
	−0.68 to 0.68	145.0–158.9	50.55	222	44,651	4.97
	0.68 to 4.5	154.7–179.4	25.11	127	22,113	5.74
50–65 (adult)	−4.5 to −0.68	154.0–173.5	24.37	88	21,386	4.11
	−0.68 to 0.68	171.5–182.0	50.77	220	45,277	4.86
	0.68 to 4.5	180.0–199.0	24.85	121	21,895	5.53

^a^The range of heights across categories is slightly overlapping, because the range is presented across birth cohorts, and *z*-scores were calculated separately for each birth cohort

**Table 3:** Hazard ratios of prostate cancer risk in adulthood per height *z*-score at age 7 and 13 years and in adulthood, unadjusted and adjusted for height *z*-scores at the ages included at the present row (*n* = 5,871, 429 cases)

Ages in model	Height *z*-score					
	7 years		13 years		50–65 years	
	HR	95 % CI	HR	95 % CI	HR	95 % CI
7 years	1.15	1.04–1.27	–	–	–	–
13 years	–	–	1.18	1.07–1.30	–	–
Adult	–	–	–	–	1.14	1.04–1.26
7 and 13 years	0.98	0.81–1.18	1.20	1.00–1.46	–	–
7 years and adult	1.09	0.94–1.27	–	–	1.07	0.93–1.24
13 years and adult	–	–	1.15	1.01–1.32	1.04	0.91–1.18
7 and 13 years and adult	0.94	0.76–1.17	1.20	0.99–1.45	1.06	0.91–1.23

*HR* hazard ratio, *CI* confidence interval

All analyses are stratified by birth cohort (1930–1934, 1935–1939, 1940–1944, 1945–1947)

When adjusted for adult height, height at age 7 years was no longer significantly associated with the risk of prostate cancer. In contrast, the association between height at age 13 years and the risk of prostate cancer was only minimally influenced, and it remained significant even after adjustment for adult height (Table 3). Adjustment for childhood height, in particular height at 13 years, attenuated the association between adult height and prostate cancer, which in these models represent change in height from childhood to adulthood, and it became nonsignificant (Table 3).

Of the total effect of height at 7 years, the estimated proportion mediated through adult height was 38.5 % (95 % CI −50.1 to 201.3); the wide CI indicates that anywhere from 0 to 100 % is mediated through adult height. The estimated mediated proportion of height at 13 years was 15.6 % (95 % CI −43.4 to 93.7) of the total effect; the wide CI indicates that anywhere from 0 % to a large proportion is mediated through adult height.

The effect of change in height from 7 to 13 years of age on the risk of prostate cancer was examined by including both height measurements in the same model. Height at 13 years, conditioned on initial height at age 7 years, which represents excess growth from 7 to 13 years of age, was positively and borderline significantly associated with prostate cancer risk (HR_13years_ = 1.20, 95 % CI 1.00–1.46) (Table 3). When we additionally included adult height in this model, the association with height at 13 years, conditional on both height at 7 and adult height, remained of the same strength as compared to the models including height at each age separately or height at both 7 and 13 years (Table 3). The estimate for height at 13 years, conditional on heights at 7 years and in adulthood, represents the timing of growth as it compares men with different heights at 13 years but who had the same heights at age 7 years and as adults.

## Discussion

We found positive and significant associations between childhood and adult height, respectively, and the risk of prostate cancer. Interestingly, we found that height at 13 years remained significantly and positively associated with the risk of prostate cancer independently of adjustment for adult height meaning that the association between height at 13 years and the risk of prostate cancer was not entirely mediated through adult height. Our results therefore indicate that being tall at 13 years of age, at least to some extent, increases the risk of prostate cancer independently of attained adult height.

Height at 13 years may be a biological marker for an increased level of insulin-like growth factor 1, which is especially high during puberty [14] and is associated with an increased risk of prostate cancer [15]. It is, however, also possible that height at 13 years is an indicator for a risk established by an unmeasured third factor that operated earlier in life and increases height during this late part of childhood as well as the risk of prostate cancer. This may explain why height at 13 years was not entirely mediated through adult height and hence seems to affect prostate cancer risk through different pathways than adult height. From the results of the present study, we cannot conclude whether the effect of height at 7 years is due to a direct effect or whether it is mediated by adult height.

In the model including height at 7 and 13 years, height *z*-score at 13 years estimates the effect of a greater growth in height from 7 to 13 years. The results indicated that growth from 7 to 13 years of age was more important than height at 7 years conditional on height at 13 years for the risk of prostate cancer. When heights at 7 years, at 13 years, and in adulthood were all in the same model, only the estimate for height at 13 years remained positive and significant. The interpretation of this estimate is that a child who had an “average” height at 7 years and in adulthood, but was tall at 13 years, has an increased risk of prostate cancer compared to a child who had an “average” height at all three points in time.

The correlations between height at 7 years and adult height (*r* = 0.77) were broadly similar, albeit slightly stronger than at age 13 years (*r* = 0.70). A possible explanation for this may be that age at peak height velocity affects the correlation between height at 13 years and adult height. In a study of the 1958 British birth cohort, correlations among boys between height at ages 7, 11, and 16 years, respectively, and age 33 years were *r* = 0.70, *r* = 0.72, and *r* = 0.77 [16]. The slight differences between these correlations and those from the current study may be explained by the different ages at measurement or random error.

Two previous studies investigated the association between childhood height and risk of prostate cancer. One small study including 33 prostate cancer cases reported an odds ratio of 1.10 (95 % CI 0.80–1.51) [17] for height at age 2–14 years. In our previous study including 3,355 cases among men from the CSHRR, the HR was 1.13 (95 % CI 1.09–1.17) [18] per height *z*-score at 13 years. Thus, the results in the present study on a subsample of the CSHRR and our previous study are largely consistent.

Additionally, numerous studies have reported a positive association between adult height and the risk of prostate cancer [3, 19–24]. A meta-analysis including 31 cohort studies reported an odds ratio of 1.09 (95 % CI 1.06–1.12) for the risk of prostate cancer per 10-cm increment in height [4], which is very similar to the effect of adult height in the current study (HR 1.14, 95 % CI 1.04–1.26).

We did not identify other studies that reported on associations among childhood height, adult height, or other potential mediators and prostate cancer. Consideration of other potential mediating pathways is compromised by the limited knowledge of the origins of prostate cancer. Evidence is emerging that metabolic risk factors in adulthood are associated with the risk of prostate cancer, but results are inconsistent [21, 24, 25]. Therefore, mediation of the effect of childhood height through metabolic factors is likely of low importance. It remains a possibility that childhood height is not mediated by measurable factors expressing their effects on prostate cancer in adulthood, but leaves a permanent effect that is initiated already during childhood.

In this study, we are examining incident prostate cancer rather than mortality or survival. The distinction is important as many forms of prostate cancer are indolent and unlikely to progress to death. Adult height is particularly associated with aggressive and fatal forms of prostate cancer [4, 26, 27]. In a previous study, we found that childhood height was positively associated with both prostate cancer death and survival [28]. Due to the low number of prostate cancer deaths in this population, we were unable to examine survival in this study.

The major strength of this study is the combination of data sources providing information on measured height all the way from childhood to adulthood in an ethnically homogeneous population combined with the long-term follow-up in the Danish Cancer Registry. As heights were measured both in childhood and adulthood, they are not subject to recall bias. Any misclassification from these measurements is most likely nondifferential, potentially leading to bias toward the null.

The study limitations include the relatively small number of cases, and that information on possible childhood height determinants, such as parental socioeconomic position, genetics, dietary status, and onset of puberty, was not available [14, 15]. Further, as Gleason score information was not electronically available, we were unable to examine associations between height and the prostate cancer aggressiveness. As for puberty assessment, no single measure exists for the onset of puberty in boys that can be ascertained with reasonable accuracy of recall [14]. The participation rate in the DCH cohort was 34 %, and the participants had a higher socioeconomic position than nonparticipants [8]. Attrition in the present study resulted mainly from a lack of identification of DCH participants in the CSHRR, which was dependent on schooling in Copenhagen. Thus, the generalizability of our results may be reduced. In addition, the included study sample was slightly taller (0.0–0.5 cm across birth cohorts and ages) than all boys from the CSHRR born in the years 1930–1947. Therefore, a follow-up selection bias cannot be excluded. Our findings need to be confirmed in larger samples and in other settings of other ethnicities.

In conclusion, we found that height at 7 years of age increased the risk of prostate cancer only before adjustment for height at 13 years and/or adult height. In contrast, height at 13 years of age had a significant and positive association with the risk of prostate cancer that was not entirely mediated through adult height or dependent on height at 7 years. This means that being tall at 13 years of age increased the risk of prostate cancer independently of attained adult height and that height at 13 years is not just a proxy for final height. Until more evidence on the origins of prostate cancer is available, the present results encourage a search for determinants of adolescent height that create the pathway to prostate cancer.

## Abbreviations

CI: Confidence intervals
CSHRR: Copenhagen School Health Records Register
DCH: Diet, Cancer and Health
HR: Hazard ratio
LMS: Lambda, Median, Sigma
SD: Standard deviation
